# Object-Based Point Cloud Analysis of Full-Waveform Airborne Laser Scanning Data for Urban Vegetation Classification

**DOI:** 10.3390/s8084505

**Published:** 2008-08-04

**Authors:** Martin Rutzinger, Bernhard Höfle, Markus Hollaus, Norbert Pfeifer

**Affiliations:** 1 alpS - Centre for Natural Hazard Management, Grabenweg 3, A-6020 Innsbruck. E-mail: rutzinger@alps-gmbh.com; 2 Institute of Geography, University of Innsbruck, Innrain 52, A-6020 Innsbruck. E-mail: martin.rutzinger@uibk.ac.at; 3 Institute of Photogrammetry and Remote Sensing, TU Vienna, Gußhausstraße 27-29, A-1040 Vienna. Email: bh@ipf.tuwien.ac.at. E-mail: np@ipf.tuwien.ac.at; 4 Christian Doppler Laboratory “Spatial Data from Laser Scanning and Remote Sensing” at the Institute of Photogrammetry and Remote Sensing, TU Vienna, Gußhausstraße 27-29, A-1040 Vienna. E-mail: mh@ipf.tuwien.ac.at

**Keywords:** Object-based point cloud analysis, Urban vegetation, Segmentation, 3D feature calculation, Classification, Error assessment, Full-waveform, Airborne laser scanning

## Abstract

Airborne laser scanning (ALS) is a remote sensing technique well-suited for 3D vegetation mapping and structure characterization because the emitted laser pulses are able to penetrate small gaps in the vegetation canopy. The backscattered echoes from the foliage, woody vegetation, the terrain, and other objects are detected, leading to a cloud of points. Higher echo densities (>20 echoes/m^2^) and additional classification variables from full-waveform (FWF) ALS data, namely echo amplitude, echo width and information on multiple echoes from one shot, offer new possibilities in classifying the ALS point cloud. Currently FWF sensor information is hardly used for classification purposes. This contribution presents an object-based point cloud analysis (OBPA) approach, combining segmentation and classification of the 3D FWF ALS points designed to detect *tall vegetation* in urban environments. The definition tall vegetation includes trees and shrubs, but excludes grassland and herbage. In the applied procedure FWF ALS echoes are segmented by a seeded region growing procedure. All echoes sorted descending by their surface roughness are used as seed points. Segments are grown based on echo width homogeneity. Next, segment statistics (mean, standard deviation, and coefficient of variation) are calculated by aggregating echo features such as amplitude and surface roughness. For classification a rule base is derived automatically from a training area using a statistical classification tree. To demonstrate our method we present data of three sites with around 500,000 echoes each. The accuracy of the classified vegetation segments is evaluated for two independent validation sites. In a point-wise error assessment, where the classification is compared with manually classified 3D points, completeness and correctness better than 90% are reached for the validation sites. In comparison to many other algorithms the proposed 3D point classification works on the original measurements directly, i.e. the acquired points. Gridding of the data is not necessary, a process which is inherently coupled to loss of data and precision. The 3D properties provide especially a good separability of buildings and terrain points respectively, if they are occluded by vegetation.

## 1 Introduction

Airborne laser scanning (ALS) is commonly used for high resolution digital terrain model (DTM) derivation [1],[2], but became also an important tool for object classification and parameter estimation for several applications such as in forestry and in urban applications. Ongoing work in forestry focuses on the delineation of stands and single trees and their parameterization [3],[4]. However, urban areas comprise a manifold of object types, which makes delineation, classification and parameterization a challenging task. Neighboring or connected objects of different classes such as buildings and overlaying trees have always been a problem in ALS classification. Therefore, tall objects such as buildings and tall vegetation are often separated by the calculation of a normalized differential vegetation index (NDVI) integrating additional remote sensing data from optical sensors (e.g. [5],[6]). Built-up areas can also be excluded easily by using for example cadastral data. But the problematic spots where buildings are covered by trees still remain an unsolved problem in 2D. However, a classification approach as it is suggested in this paper, working directly in 3D, can solve this problem. Points classified as a rooftop may, for example, lie directly beneath points classified as belonging to a tree. This is not possible if data processing is performed in 2D with height (and other features) treated as properties (so-called 2.5D approach).

The recent developments of full-waveform (FWF) ALS provide additional attributes. These are the amplitude and the echo width, and in comparison to traditional discrete return systems provide information beyond the x, y, z coordinates. In Wagner et al. [7] initial results indicate that a differentiation of objects may benefit from those additional attributes. As a third aspect, the growing availability of high density ALS data has to be mentioned. In current ALS data acquisitions typically more than 10 points per m^2^ are collected. The developments in sensor technology, but also the prospect for solving the issue of overlapping objects, demand adaptations and extensions of existing algorithms to be able to process this new type and larger amount of data.

The concept of object-based image analysis (OBIA) has been developed in the field of digital image analysis to manage analysis of high resolution data (e.g. large scale object representation or noise suppression in classification results) [8]. To maintain the full resolution and information in ALS data and to avoid any rasterization and conversion to a 2.5D model, which also requires choosing a resolution and an aggregate function, an object-based point cloud analysis (OBPA) concept is designed to perform segmentation, feature calculation, classification, and parameter estimation within the original point cloud. This concept was already successfully applied for roof face classification [9] and glacier surface classification [10]. The algorithm presented is applied to detect urban vegetation. The classification of vegetation ALS points has several applications such as improvement of DTM generation [11], establishing or updating urban tree register (cadastre), or deriving tree models for visualization in digital 3D city models [12],[13].

Dedicated vegetation studies are often performed using data acquired under leaf-on conditions. However, multiple use of ALS point clouds and short time frames for the acquisition often lead to data sets acquired over urban areas under leaf-off or even mixed conditions. Especially for the derivation of DTMs the leaf-off acquisition is the preferred one. The data used in this study was acquired during leaf-off conditions. To the authors' best knowledge there are hardly any investigations on tall vegetation under leaf-off condition. In any case, it has to be considered that foliation conditions strongly influence parameters and strategies of vegetation classification.

In the following study, Section 2 related work on ALS and vegetation mapping is described. In Section 3 the data sets and the test site are introduced. Section 4 explains the OBPA methods comprising feature calculation, segmentation and classification and Section 5 describes the settings of the workflow. Section 6 discusses the error assessment procedure and the gained results. Finally, the presented work is concluded in Section 7 and the outlook points out future work and further improvements.

## 2 Related work

Several studies show that segmentation and classification respectively of ALS data in urban areas lead to promising results. However, most investigations work with rasterized data sets using a combination of ALS and ancillary data (e.g. infrared information from imagery or building ground plans) to separate vegetation and building objects (e.g. [14],[15]). Matikainen et al. [16] distinguish buildings, tall vegetation and terrain using an OBIA approach (cf. [17]). In a first step a last echo DTM is segmented. Next, different combinations of segment features from a first and a last echo digital surface model (DSM), the calculated difference of these two models, and an aerial image are used as raster input layers for classification. The most significant features (e.g. mean difference between first and last echo DSM, Grey-Level Co-occurrence Matrix homogeneity of the first echo DSM, or average length of segment edges) and the final rules for classification are selected by a classification tree.

The second group of investigations detects objects in the 3D point cloud using geometry features in order to outline homogeneous areas. They are defined for example by their planarity or by elevation derivatives (e.g. slope or height differences), mainly to derive building roofs and terrain patches (e.g. [18],[19]). Such approaches will fail for the rough vegetation canopy. Often vegetation points are collected in a class with other objects showing no regular geometrical behavior.

Melzer [20] segments the 3D point cloud using a mean shift approach. The advantage of this procedure is the independence on any geometrical appearance of the segmented objects or surfaces respectively. The segmentation is successfully applied to complex urban test sites as well as to power line segmentation, where approaches in 2.5D, range image segmentation, or model based segmentation approaches reach their limit.

An application of point cloud segmentation in hydrology is presented by Straatsma and Baptist [21] delineating single tree crowns and herbaceous vegetation for the parameterization of hydrodynamic roughness of floodplains. First low vegetation is excluded by a threshold on normalized heights and low number of neighbors. Herbaceous vegetation is assumed to be more sparsely growing and characterized by only few points while young trees, which could be also smaller than herbs tend to be represented as clumped points. The segmentation is then performed by *k*-means clustering using local maxima as seed points. The clustering leads to an overestimation of clusters. Enhancement is reached by analyzing 1 m wide cross sections of neighboring clusters and their maximums based on distances, height differences, and surface structure. The final segmentation result is reached by iteratively merging the clusters.

Recently research started using the additional information of FWF ALS variables in 3D space. Gross et al. [22] present an approach to detect tree regions from FWF ALS data for an urban test site. Beyond amplitude, echo width, and total number of echoes, additional ‘volumetric’ (3D) features, i.e. based on covariance such as planarity or omnivariance are calculated directly in the 3D point cloud to characterize the tree points. Two different parameter sets are tested. Using a combination of the features (mean intensity, planarity and omnivariance) calculated in a certain neighborhood leads to a lower false alarm rate while using FWF variables only (echo width, amplitude, and total number of echoes) shows a higher detection rate.

An approach using normalized heights together with FWF ALS data is presented in Reitberger et al. [23]. They delineate single trees and detect the tree positions of forest stands. After deriving normalized heights the crowns and tree stems are extracted from a smoothed normalized digital surface model (nDSM). It is shown that the consideration of the detected tree stems improves the estimation of the tree position.

ALS point cloud analysis and FWF studies mainly work on characterization of vegetation for applications in forestry. Vegetation and land cover classification studies with focus on urban areas mainly require rasterized input data. Hence, the presented work contributes to 3D classification of ALS echoes in urban areas.

## 3 Test site and data sets

### 3.1 Test site

**Figure 1.**
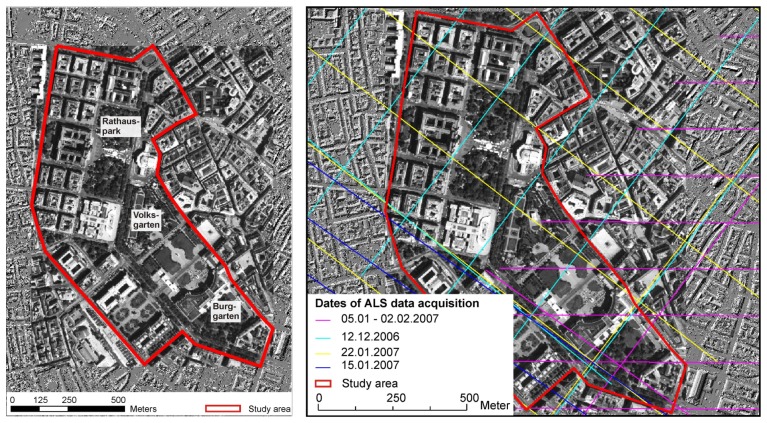
Study area (left) and nadir flight paths (right).

The developed analysis is done for three different subsets in park sites (*Rathauspark, Burggarten* and *V olksgarten*) in a 75 ha subarea of the city center of Vienna. The area is dominated by several deciduous (e.g. maple, conker, plane and lime) and coniferous tree species (e.g. Scotch pine and Norway spruce). Figure 1 gives an overview of the study area.

The site *Rathauspark* is a skirting park area dominated by full-grown deciduous trees and compact shrubs. It contains long lines of benches along the walking paths, buildings connected or occluded by vegetation, and one part of a strongly structured facade of a multistory building. Most buildings are smaller than 3 m height with flat and gabled roofs. There are also several types of parked vehicles (e.g. passenger cars, trucks).

The second investigation site (*Burggarten*) contains deciduous and coniferous trees, which differ in species and size, and compact shrubs. Additionally, it contains a conspicuous park fence and walls.

The site *Volksgarten* contains deciduous trees only. It is dominated by a large deciduous tree, which is connected to an avenue of smaller trees. Very low cut shrubs in regular line patterns are distinctive for this site. It contains artificial objects such as ventilation shafts, which are covered by an iron grid, and a fountain (filled with water and with four islands of tall grass) surrounded by a small wall. Furthermore, it contains a single, free-standing building, built in Greek-style, which means the building walls are not solid but columns with a gabled roof on top.

### 3.2 Full-waveform ALS data

The available FWF ALS data are provided by the city of Vienna (MA41-Stadtvermessung^1^). The city-wide ALS project was carried out during the winter and spring season 2006/2007. It has to be considered that the vegetation was scanned in leaf-off conditions, which influences the reflectance properties of deciduous plants. The data were acquired by the company Diamond Airborne Sensing GmbH^2^ in cooperation with AREA Vermessung ZT GmbH^3^ using a RIEGL LMS-Q560^4^ full-waveform scanner. The LMS-Q560 uses short laser pulses with a wavelength of 1.5 *μm* and a pulse width of 4 ns. The laser beam divergence is 0.5 mrad and the scan angle varies between ±22.5°. The flight strips have a crosswise overlap of approx. 50%. The average flight altitude was about 500 m above ground, which resulted in a theoretical laser footprint diameter of 25 cm on ground. Within the study area the mean point density varies between 15 and 20 emitted laser pulses per m^2^. The relevant sensor characteristics of the applied ALS system are summarized in Table 1.

In comparison to traditional discrete ALS systems, where only single echoes are recorded, the full-waveform system RIEGL LMS-Q560 records the entire parts of the backscattered waveform whose amplitudes exceed a threshold that is defined by the system. Thus, only data containing information on detected targets are transmitted to a data recorder, which leads to tremendous data reduction without loss of information.

Wagner et al. [24] found that the system waveform of the used RIEGL LMS-Q560 can be described by a Gaussian function. Therefore, the backscattered waveform is a result of a convolution of the system waveform and the differential cross section of the illuminated object surface. Based on a Gaussian decomposition individual targets can be detected within a waveform, whereas the range, the amplitude, and the width are obtained for each echo. Based on the short pulse duration of 4 ns the range resolution is 0.6 m. Consequently, distinct echoes are produced if neighboring targets are separated by distances larger than the range resolution, while the number of detectable echoes per laser shot is not limited. The echo width provides information on roughness within the laser footprint, the slope of the surface, or the depth of a volumetric target [25]. As shown in Wagner et al. [7] the echo width is a useful quantity for deciding whether a pulse was reflected from a solid surface (i.e. terrain, roof) or from vegetation and therefore improves the classification of the last echoes into terrain and off-terrain points. If a backscattering target is larger than the laser footprint the amplitude of the echo can be directly related to the target's reflectance. According to Wagner et al. [24] an adapted radar equation can be applied to convert the received power (i.e. expressed as amplitude and echo width) into the backscatter cross section. As the backscatter cross section represents a calibrated physical quantity, it is comparable if the measurements are done with different ALS systems.

### 3.3 Reference data

The evaluation of classification results in many remote sensing studies is disturbed by inadequate reference data not representing exactly the same objects of interest. Therefore, in this investigation the 3D point cloud is filtered manually^5^, which is supported by extensive field investigations and detailed photographs for problematic areas. Beside errors, which could be made by the interpreter all other errors measure the detection success of the applied workflow. They are free from influences such as differences in resolution, date of collection, data source, or data model. A detailed view of the reference data gives Figure 6.

The area *Rathauspark* is used as training area to derive the settings of the OBIA workflow, while the other two areas *V olksgarten* and *Burggarten* are the validation sites. Around 500,000 echoes were recorded in each test site. These points are classified manually using the DTMaster Software (INPHO Gmbh^6^). The classes comprise terrain points, buildings, vegetation and other tall objects (e.g. benches, fences, cars, or outliers). In order to compare the OBPA classification results with the manually classified reference points the classes were merged into tall vegetation and non-vegetation. The definition *tall vegetation* includes trees and shrubs, but excludes grassland and herbage lower than 20 cm.

## 4 Methods of the object-based point cloud analysis workflow

**Figure 2.**
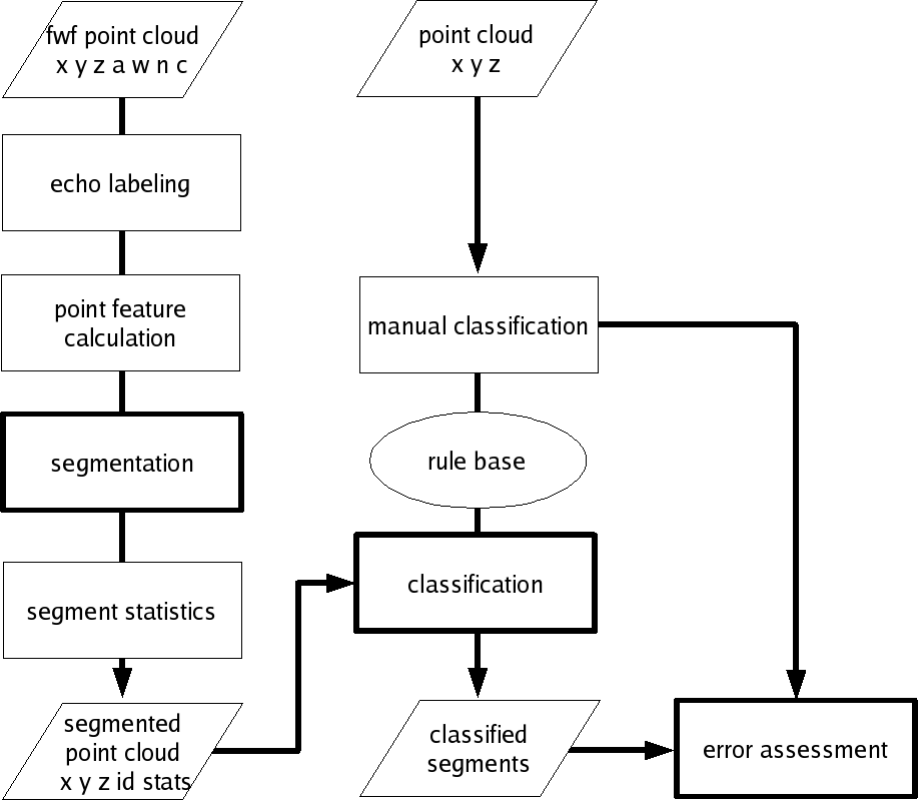
Workflow of object-based point cloud analysis for vegetation detection (a = amplitude, w = echo width, n = echo number, c = echo count, id = segment id, stats = segment statistics).

For the detection of urban vegetation the concept of OBPA is applied, which is a combination of point feature calculation (Sect. 4.1), segmentation (Sect. 4.2), classification (Sect. 4.3) and error assessment (Sect. 4.4) using solely the ALS point cloud (Fig. 2). A preliminary study on feature calculation and segmentation implementation on pulsed ALS data for urban vegetation is described in Rutzinger et al. [26]. Based on this foregoing study the following method was further developed and adapted to FWF ALS data.

### 4.1 Additional point features

Urban objects can be parameterized by several features in order to distinguish vegetation and other objects such as buildings, cars or terrain points. FWF ALS data offers the possibility to describe objects by their geometry (x, y, z coordinates) as well as by their reflectance property (amplitude) and the target roughness within the footprint (echo width). Vegetation is characterized by a geometrical inhomogeneity, which can be parameterized by for example roughness or a point density ratio measure [26]. Amplitude calibration [27],[28] is not carried out since only low effects due to scan geometry and atmosphere are present in the selected subsets. Very high amplitude values caused by specular reflectance have been excluded to improve the stability of the calculated segment statistics. Furthermore, the echo width, which is insensitive to the flight geometry is used for both, segmentation and classification.

The spatial neighborhood required for feature calculation can be determined either in 2D (a circle projected on a horizontal plane) or 3D (spherical) domain. The neighborhood selection is either done by *k*-nearest neighbors (selecting a defined number of nearest neighbors) or by using a fixed distance search radius *r* [m]. The neighborhood definition is a function of the object size and the point density. Here a radius of 0.5 m is used for all features applying a fixed distance neighborhood definition.

Surface roughness can be expressed using several parameters such as the standard deviation (SD) of z-values or the SD of the plane fitting residuals in the fixed distance 3D neighborhood. The latter is used for the current analysis. It helps to distinguish smooth surfaces, such as building roofs and terrain points, from trees and shrubs, characterized by high roughness values. While building walls and inclined roof faces have a high SD of z-values, the here defined roughness will characterize them as smooth surfaces which will contribute to a better differentiation of inclined or vertical plane surfaces and the rough vegetation canopy.

A second feature to describe vegetation is the point density calculated in both, 2D *p*_2_*_D_* [m^−2^] (Eq. 1) and 3D *p*_3_*_D_* [m^−3^] (Eq. 2) domain. The investigation of point density calculated in 3D (*p*_3_*_D_*) shows a high separability of penetrable (mainly vegetation points) and solid surfaces. However, *p*_3_*_D_* suffers from disturbing artifacts caused by overlapping flight strips and flight geometry distortions such as heading or pitch. The calculation of a point density ratio (*DR*_3_*_D_*_/2_*_D_*) [m^−1^] suppresses these effects (Eq. 3).

(1) $$p_{2D}=\frac{N_{2D}}{\pi *r^{2}}$$

(2) $$p_{3D}=\frac{N_{3D}}{\frac{4}{3}*\pi *r^{3}}$$

(3) $$DR_{3D/2D}=\frac{p_{3D}}{p_{2D}}=\frac{N_{3D}}{N_{2D}}*\frac{3}{4*r}$$

In addition to the echo width the occurrence of intermediate echoes is a valuable feature to describe tall vegetation, because the height difference is large enough that multiple reflections occur within one laser beam. The raw data points are delivered with attributes on total number of echoes (*N_e_*) and echo count (*C_e_*) per shot. They are used to classify single (*N_e_* = 1 AND *C_e_* = 1), first (*N_e_* > 1 AND *C_e_* = 1), intermediate (*N_e_* > 1 AND *C_e_* > 1 AND *N_e_*! = *C_e_*), and last echo (*N_e_* > 1 AND *N_e_* = *C_e_*). A ratio (‘echo ratio’) between the sum of first and intermediate echoes to all single echoes is calculated for every point in a 3D fixed distance neighborhood (Eq. 4).

(4) $$\text{echo rati}o^{7}=\frac{N_{\text{e first}}+N_{\text{e intermediate}}}{N_{\text{e single}}}$$

### 4.2 Segmentation

The implemented algorithm is a seeded region growing segmentation using all points sorted descending by their roughness as seed points. The region growing itself uses the echo width to group the laser points into segments. For each seed point *k*-nearest neighbors are selected as candidate points, which are checked according to their echo width. The homogeneity is defined as an indirect proportional criterion calculated from the user set tolerance and the echo width of the starting seed point. For example a user tolerance of 1 ns at a starting seed point with 4 ns echo width results in a homogeneity tolerance for growing of ±0.25 ns; a starting seed point with 2 ns echo width will have a homogeneity tolerance of ±0.5 ns. If the candidate point lies within the defined maximum deviation to the seed echo width it becomes part of the segment and is used as a next seed point. The indirect proportional homogeneity is a very sensitive measure if the echo width is high, which is the case for vegetation echoes. This will result in rather small segments but it ensures a more accurate definition of the boundaries between vegetation and non-vegetation segments. The growing of the segment is limited by a 3D maximum growing distance [m] and the segment size (minimum and maximum number of points per segment). Finally, for each segment basic statistics such as number of points, minimum, maximum, SD, mean and coefficient of variation (*Cν*) based on the available point features (Sect. 4.1) are calculated.

### 4.3 Classification tree

**Figure 3.**
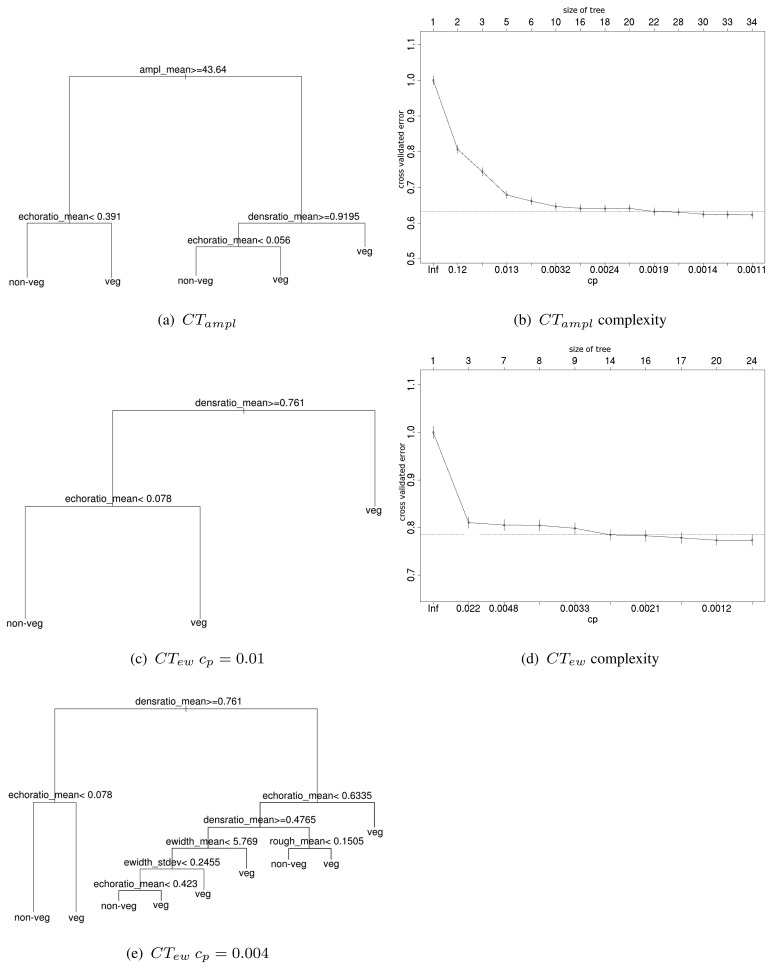
Derived classification trees for training site *Rathauspark* using either echo width or amplitude as additional feature. The error diagrams show the cross validated error plotted against classification tree size for changing complexity parameter settings.

For the separation of vegetation and non-vegetation a set of rules is applied (Fig. 3), which is derived automatically by a classification tree (CT). The decisions in the rule base are formulated on the derived segment features. The segment features are compared against the reference data in a training site in order to derive an optimized rule base. CTs follow an iterative approach. First the variable is determined splitting the data best into two groups. Then this is done for all subgroups recursively until either a minimum size of the subgroup is reached or no further improvement can be made. Additionally, the user can control the size (=number of splits and hierarchy levels respectively) of the CT by defining the complexity parameter (*c_p_*). The *c_p_* stands for the cost, which is added for every split and hence, controls the total number of splits. A high *c_p_* would lead to a smaller tree, while a low *c_p_* would produce more branches and a more complex tree. In general, complex trees are difficult to interpret because the decisions are derived data driven. For a training data set all features per segment are tested and the data is partitioned into the final classes (recursive partitioning). In the final classification not all, but only the most prominent features are used [29],[30].

The used CT algorithm is implemented in the *rpart* package [31],[32] of the statistical software R [33]. The CT is a hierarchical structure starting at a root node. All nodes are connected by non-terminal nodes, where the belonging to a class is decided. If the condition at a node is fulfilled, the branch to the left is followed. The splitting criterion is a diversity index, which decides on whenever splitting should stop or not. For each splitting criterion the error is measured by a modified version of the Gini index (Eq. 5).

(5) $$\sum_{j\neq k}p_{ij}p_{ik}=1-\sum_{k}p_{ik}^{2}$$

A certain number of observations *n_ik_* (segments) is assigned to the class *k* at the *i^th^* leaf of the classification tree. *n_ik_* estimates the proportions *p_ik_*. Each leaf is a potential candidate to become a new node for splitting.

The cross validated error is calculated in *rpart* by dividing the training data into *m* subsets. The CT is modeled for *m*-1 subsets, while the left out one is used for validation. The cross validated error is plotted against *c_p_*, which controls tree complexity (see Fig. 3(d)). The plot is a tool to analyze the tree structure in order to keep errors and tree complexity at a minimum.

### 4.4 Error assessment

The workflow in Figure 2 is applied to the training area in order to tune the segmentation settings and to derive the CT. The error assessment informs about the applicability of the workflow within this area. Then the procedure designed for the training area is applied to the two independent validation sites. For the error assessment completeness (*Comp*) and correctness (*Corr*) are calculated (Eq. 6 and Eq. 7).

(6) $$\text{Comp}=\frac{\left\|TP\right\|}{\left\|TP\right\|+\left\|FN\right\|}$$

(7) $$\text{Corr}=\frac{\left\|TP\right\|}{\left\|TP\right\|+\left\|FP\right\|}$$

The classes vegetation and non-vegetation are compared by the class label of each laser echo in the reference and classification data set. The corresponding echoes are defined by the comparison of the x, y, z coordinates between both data sets, where a true positive (*TP*) has identical labels in both data sets, false negative (*FN*) is labeled in the reference data but has no correspondence echo in the classification, and false positive (FP) is labeled in the classification and has no corresponding label in the reference data [34]. The overall accuracy is the *Comp* calculated for all classes in the reference data set, while the average accuracy is the average *Comp* for all classes.

## 5 Object-based point cloud analysis settings

### 5.1 Segmentation settings

Most reliable segmentation results, which should guarantee the separation of objects of interest by the grouped points, were achieved by applying the settings in Table 2. The selection of the segmentation settings (especially of the *k*-nearest neighbors and the 3D maximum growing distance) are strongly dependent on the point distribution and point density. The segmentation itself runs on homogeneity of the echo width, which shows high separability of vegetation and other objects. The seed points are all points sorted descending by their roughness value, which is defined as the SD of the orthogonal plane fitting residuals within a fixed distance in a 3D neighborhood (Sect. 4.1). As found in Rutzinger et al. [26] it can be assumed that vegetation points have a higher roughness value than others and therefore they are good estimates to start growing regions.

### 5.2 Classification tree settings

The CT is fed with the segment statistics (mean, SD, and *Cν)* based on the features amplitude, echo width, roughness, density ratio, and echo ratio (as described in Sect. 4.1). CTs are modeled for site *Rathauspark* using (i) all features (*CT_all_*), (ii) leaving out amplitude features (*CT_ew_*) and (iii) leaving out echo width features (*CT_ampl_*) in turn. Tests on *c_p_* are made in order to include and exclude features in the CT.

The variation of the input features for *Rathauspark* shows that with *c_p_*=0.001 the echo with is not included in the classification tree if using all available features. If *c_p_*=0.01 the rules of *CT_all_* are equal to *CT_ampl_*. *CT_ampl_* is stable in the range of *c_p_* 0.01 to 0.006 (Fig 3(b)), since splitting will not change. Every additional split and reduction of *c_p_*, respectively, would only lead to a minimal further reduction of the cross validated error. If amplitude (*CT_ew_*) is excluded, the tree becomes less complex, but the echo width is still not used as splitting feature. If *c_p_* gets lower than 0.004 echo width is included, but only in branches containing a marginal amount of points (Tab. 3). In general, *CT_ew_c_p_*=0.004 seems to be ‘over-fitted’. This is also evident by the minimal changes of the cross validated error in Figure 3(d) by increasing *c_p_* and the number of splits respectively.

The rules of most modeled trees work on mean segment features. This shows that vegetation and non-vegetation segments are characterized best by homogeneity and not by high deviations within the segments, which would be described by SD or *Cν*. The SD of echo width is used only for three branches in *CT_ew_c_p_*=0.004.

## 6 Results and discussion

### 6.1 Segmentation

The implemented indirect proportional homogeneity criterion (Sect. 4.2) leads to large segments for non-vegetation objects because they are characterized by lower footprint roughness (echo width) than tall vegetation. Since vegetation is very irregularly structured it is an advantage that vegetation segments tend to be smaller, keeping segment statistics undistorted. The decision to skip the minimum segment size is drawn to keep all points as long as possible in the point processing workflow. Smallest segments tend to an ambiguous feature representation, because they are susceptible to outliers, if they only consist in for example 5 or less points. This was taken into consideration, because it is better to have a failure on small segments in classification than to drop them immediately and produce lower error rates. The introduction of a merging procedure on smallest segments could not improve the results. It is supposed to merge those segments to the neighboring larger one, which lead to the merging of stem and shrub segments together with terrain segments. The application of a height dependent merging allowing only merging if the larger neighboring segment has a higher z-value could not clear up the ambiguity that segments of different objects of interest got merged. Therefore, smallest segments remained for classification and the final classification result is enhanced by a mode filter on class ID instead.

### 6.2 Interpretation of classification tree branches

**Figure 4.**
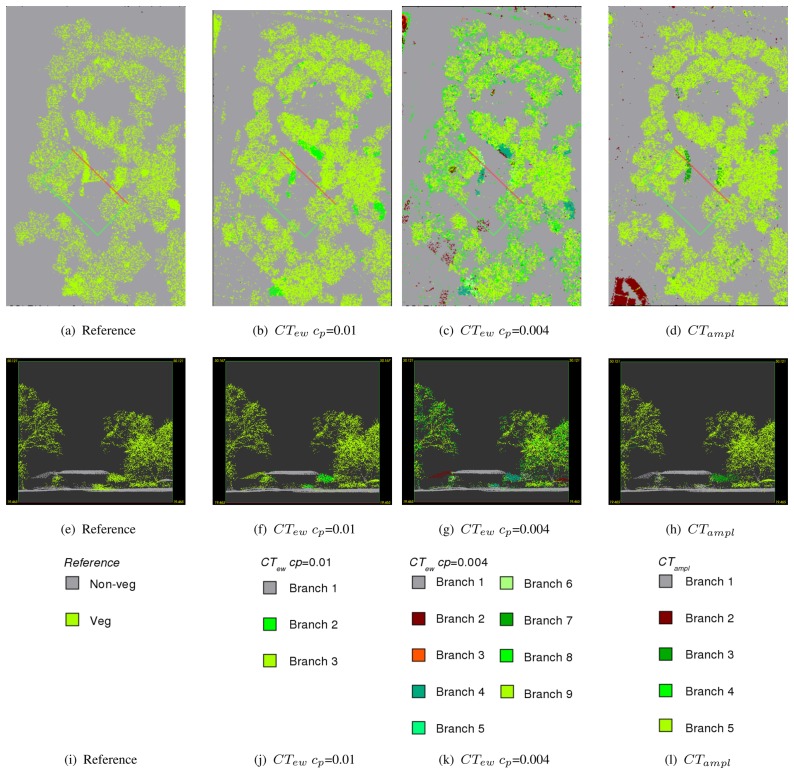
Sample profiles showing reference, *CT_ew_ c_p_*=0.01, *CT_ew_ c_p_*=0.004, and *CT_ampl_* colored by end nodes (=branches) for test site *Rathauspark*.

**Figure 5.**
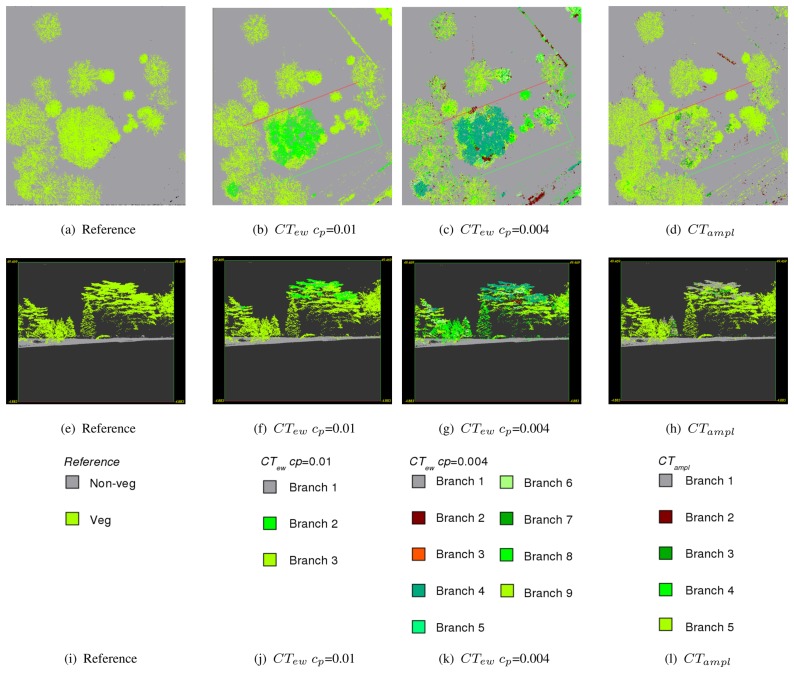
Sample profiles showing reference, *CT_ew_ c_p_*=0.01, *CT_ew_ c_p_*=0.004, and *CT_ampl_* colored by end nodes (=branches) for the validation site *Burggarten*.

**Figure 6.**
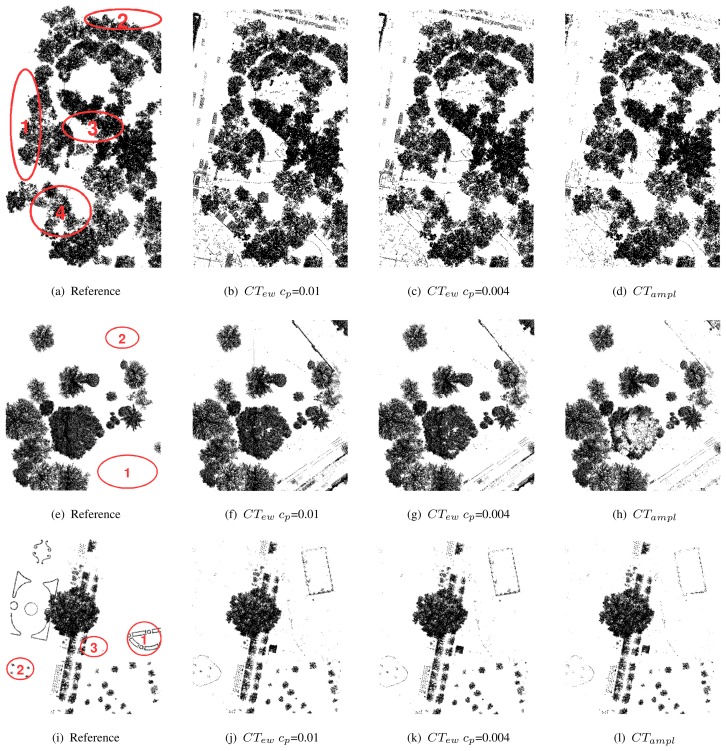
Comparison of classified vegetation points in reference, *CT_ew_ c_p_*=0.01, *CT_ew_ c_p_*=0.01 and *CT_ampl_* for the three sites *Rathauspark* (a-d), *Burggarten* (e-h), and V *olksgarten* (i-l). In (a) areas with parking cars are labeled as 1 and 2. 3 and 4 are examples of buildings connected or covered by trees. In (e) areas with park fences and walls are labeled as 1 and 2. In (i) areas with short cut vegetation are labeled as 1, the fountain with grass islands as 2, and the ventilation shaft as 3.

Table 3 shows all CTs modeled for the training area *Rathauspark*. It shows the single branches, the rules, the belonging class, and the percentage of echoes within each branch for all sites. All CTs have a predominant vegetation and non-vegetation branch containing a major part of the echoes in all investigated sites. The most important features to split vegetation from non-vegetation are *density ratio_mean_* and *echo ratio_mean_* if excluding amplitude (*CT_ew_*). If amplitude is available (*CT_ampl_*) *amplitude_mean_* is used instead of *echo ratio_mean_*. The comparison of the branches and their rules (Tab. 3) with the reference point cloud (Fig. 4 and Fig. 5) gives an overview of kinds of objects belonging to the branches. The predominant non-vegetation branch always contains terrain points and terrain near objects, while the predominant vegetation branch includes tree crowns and stems. The behavior of the other branches containing the minor part of the echoes is very specific for each CT. These branches comprise (i) building objects and cars, which are included in the non-vegetation class or (ii) compact shrubs, stems or parts of coniferous trees, if belonging to the vegetation class. *CT_ew_* has problems to separate buildings and vegetation, and also most cars occur within the vegetation class. These problem areas are marked in Figure 6(a) as 1 and 2 for echoes on cars and as 3 and 4 for echoes on buildings connected or overlaid by trees. The extended tree (*CT_ew_c_p_*=0.004) can sort out some of these buildings in branch 2 defined by low *echo ratio_mean_* and *echo width_mean_* (Tab. 3). However, ambiguity remains for most cars and vegetation (branch 8), and buildings and shrubs (branch 4). These problems of class definition are solved, if amplitude is available (*CT_ampl_*). Then branch 1 contains again most terrain, car, and building echoes. Branch 2 separates further building objects defined by high *density ratio_mean_* and low *echo ratio_mean_*. Compact shrubs are defined in branch 3 by high *amplitude_mean_* and high *echo ratio_mean_*.

**Figure 7.**
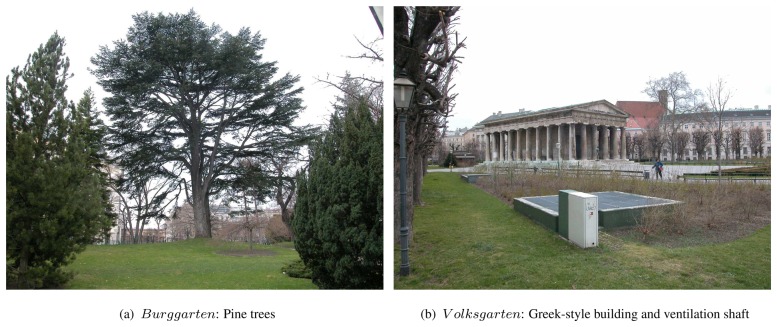
Validation sites *Burggarten* and *V olksgarten*.

Misclassification occurs at the pine tree (Fig. 7(a)) in the validation site *Burggarten*. Most segments of the upper canopy are characterized by high *amplitude_mean_* and low *echo ratio_mean_*. Therefore, they are classified as non-vegetation by *CT_ampl_*. The same phenomenon can be seen at two small conifers in the back (Fig. 5(h)). Both versions of *CT_ew_* classify the canopy of the pine tree correctly as vegetation, but it has to be considered that branch 2 (*CT_ew_c_p_*=0.01) as well as branch 4 (*CT_ew_c_p_*=0.004) have weak reliability on class definition. They include several non-vegetation objects (see above *Rathaus*). *CT_ew_c_p_*=0.004 additionally classifies some pine tree parts as non-vegetation (branch 2). For all CTs it is difficult to separate park fences and walls from vegetation. However, *CT_ampl_* classifies them best (Fig. 5(d)). These areas are marked with 1 and 2 in Figure 6(e).

The low cut vegetation, planted in regular patterns (Fig. 6(i)/1) in site *V olksgarten* cannot be detected by any CT. It is characterized by low *echo ratio_mean_* and slightly too high *density ratio_mean_* and is therefore always treated as non-vegetation (terrain). Further failure occurs for the ventilation shafts (Fig. 6(i)/2 and Fig. 7(b)) and the small wall of the fountain (Fig. 6(i)/3), which are classified as vegetation by all CTs. While the roof of the Greek-style building is always classified correctly, the columns are contained by branches belonging to the vegetation class. In this case *CT_ampl_* performs best and classifies the major column parts as non-vegetation.

In general, the investigation of the single end nodes (=branches) of the CT shows better matching to the real world phenomena and objects if amplitude is considered in the rule base. The higher *c_p_* value leads to more specific CTs which will suffer from robustness and transferability.

### 6.3 Error assessment on the vegetation class

The error assessment is done by a point wise comparison of the manually classified laser points (Sect. 3.3). This has the advantage that error values are not influenced by any other uncertainties (e.g. accuracies from the ALS measurements or ground truth collection). Hence, it only measures the failure of the applied method. The final vegetation class is established by merging all branches of the CT belonging to this class. The results for all CTs are visualized together with the manually classified reference point cloud in Figure 6. As already mentioned all CTs work very well separating terrain and terrain near vegetation echoes, which lead to high accuracies in all sites (Tab. 4).

Improved results for visual representation of the vegetation class can be achieved by applying a mode filter (1 m search radius) to the final vegetation class, which merges small isolated groups of points with the dominant class.

It was suggested that the mean statistics are strongly dependent on outliers. Therefore, the removal of outliers, based on available echo features in the 3D point cloud, would help to derive a more robust classification tree. This would enhance the transferability of the classification rule base to other data sets. As shown in Table 4 the number of outliers is very small. No significant influence on the accuracies could be found.

All tested CT settings reach accuracies over 90% (except *CT_ampl_* in *Burggarten* with 84.07% completeness for vegetation). On the one hand *CT_ampl_* computes the highest correctness values. On the other hand highest completeness is produced by *CT_ew_*. These results show an improvement in comparison to Ducic et al. [35] achieving 88.6% overall accuracy, where a CT using amplitude, echo width, and number of echoes is applied directly to the FWF echoes. In the study of Gross et al. [22] a detection rate of around 0.65 at a false alarm rate of 0.18 is reached using amplitude and additional volumetric features for vegetation classification (see Sect. 2). They found that using features considering a neighborhood (averaged amplitude, planarity, and omnivariance) within a search radius lead to higher detection and lower false alarm rate than using FWF variables (amplitude, echo width, and total number of echoes) only.

## 7 Conclusion

The presented object-based point cloud (OBPA) workflow works directly in the original 3D point cloud. The derived point features from full-waveform (FWF) information (geometric and physical quantities) are used. Hence, no additional information such as object heights is needed. High degree of automation and fast computation of classification is reached. Single echoes are grouped to segments, which are used as input in the automatically produced classification tree. The applied region growing segmentation, which uses an echo width homogeneity criterion, produces small segments in the vegetation canopy with reasonable segment boundaries between the objects of interest. Good results are produced for separating tall vegetation from non-vegetation echoes in urban areas.

The derived classification trees (CT) are very sensitive to the available segment features and the complexity parameter (*c_p_*). If the structure of the CT becomes too complex the derived rules become more site specific and suffer from less transferability and robustness. The investigation of the single branches of the rule base gives further insight into the class description by the 3D features. It can be shown that the FWF variables are valuable features for vegetation classification and especially amplitude has major importance for the definition of proper classes. It is confirmed that vegetation is characterized by low amplitude and low density ratio (*DR*_3_*_D_*_/2_*_D_*) values.

The different behavior of the classification at deciduous trees and the separation of dense shrubs and some tree stems makes it clear that the classification method and its input variables have further potential to distinguish different vegetation types (trees and shrubs), species (coniferous and deciduous trees) and tree parts (canopy, branches, and trunk). Moreover the behavior of vegetation types regarding the ALS FWF variables amplitude and echo width should be investigated under different phases of the plant phenology.

For operational use of the OBPA vegetation classification a combined OBIA and OBPA approach is suggested to take the advantage of (i) computation performance in raster analysis and the possibility of (ii) tighter classification rule definition, if a vegetation mask from OBIA is available (cf. [9]). Further investigation of the FWF sensor variables and the use of additional 3D features, such as information on missing points (i.e. laser shots with no recorded echo) [36], could offer (i) better characterization of vegetation parts and vegetation species as well as (ii) more advanced object differentiation in terms of a land cover classification in urban areas.

## Figures and Tables

**Table 1.** Specification of RIEGL LMS-Q560^4^.

Parameter	Value
Measurement range	30 m - 1800 m at target reflectivity of 60% 30 m - 1200 m at target reflectivity of 20%
Ranging accuracy	20 mm
Multi-target resolution	down to 0.5 m
Measurement rate	240,000 measurements / sec (burst rate) up to 160,000 measurements / sec (average)
Scan range	45° (up to 60°)
Scan speed	up to 160 lines / sec
Time stamping	resolution 1 *μs*, unambiguous range > 1 week
Laser safety	laser class 1, wavelength near infrared

**Table 2.** Region growing settings using echo width as homogeneity criterion.

Parameter	Settings

Seed criterion (roughness)	All points, descending
Growing criterion (echo width)	1 ns (controls dynamic tolerance depending on echo width of starting seed point)
Nearest neighbors (k)	5 points
3D maximum growing distance (dist)	0.5 m
Minimum segment size (minArea)	1 point
Maximum segment size (maxArea)	100,000 points

**Table 3.** Rules of CT split into single branches (=end nodes) derived for *RP* (= training area *Rathauspark*), *BG* (= validation site 1 *Burggarten*), and V G (= validation site 2 V *olksgarten*)

CT	End nod with Class	SQL WHERE rule	Amount of echoes [%]		
			*RP*	*BG*	*VG*

*CT_ew_ c_p_*=0.01	branch1: non-veg	density ratio*_mean_* >= 0.761	65.29	86.17	67.48
		echo ratio*_mean_* < 0.078			

	branch2: veg	density ratio*_mean_* >= 0.761	1.03	0.18	5.27
		echo ratio*_mean_* >= 0.078			

	branch3: veg	density ratio*_mean_* < 0.761	33.68	13.65	27.25

*CT_ew_ c_p_*=0.004	branch1: non-veg	density ratio*_mean_* >= 0.761	65.29	86.17	67.48
		echo ratio*_mean_* < 0.078			

	branch2: non-veg	density ratio*_mean_* < 0.761	0.98	0.13	0.96
		echo ratio*_mean_* < 0.6335			
		density ratio*_mean_* >= 0.4765			
		echo width*_mean_* < 5.769			
		echo width*_SD_* < 0.2455			
		echo ratio*_mean_* < 0.423			

	branch3: non-veg	density ratio*_mean_* < 0.761	0.07	0.02	0.06
		echo ratio*_mean_* < 0.6335			
		density ratio*_mean_* < 0.4765			
		roughness*_mean_* < 0.1505			

	branch4: veg	density ratio*_mean_* >= 0.761	1.03	0.18	5.27
		echo ratio*_mean_* >= 0.078			

	branch5: veg	density ratio*_mean_* < 0.761	0.01	0	0.12
		echo ratio*_mean_* < 0.6335			
		density ratio*_mean_* >= 0.4765			
		echo width*_mean_* < 5.769			
		echo width*_SD_* < 0.2455			
		echo ratio*_mean_* >= 0.423			

	branch6: veg	density ratio*_mean_* < 0.761	0.98	0.06	1.82
		echo ratio*_mean_* < 0.6335			
		density ratio*_mean_* >= 0.4765			
		echo width*_mean_* < 5.769			
		echo width*_SD_* >= 0.2455			

	branch7: veg	density ratio*_mean_* < 0.761	0.18	0.02	0.11
		echo ratio*_mean_* < 0.6335			
		density ratio*_mean_* >= 0.4765			
		echo width*_mean_* >= 5.769			

	branch8: veg	density ratio*_mean_* < 0.761	10.13	2.05	6.65
		echo ratio*_mean_* < 0.6335			
		density ratio*_mean_* < 0.4765			
		roughness*_mean_* >= 0.1505			

	branch9: veg	density ratio*_mean_* < 0.761	21.32	11.37	17.52
		echo ratio*_mean_* >= 0.6335			

*CT_amplitude_*	branch1: non-veg	amplitude*_mean_* >= 43.64	67.07	86.31	73.72
		echo ratio*_mean_* < 0.391			

	branch2: non-veg	amplitude*_mean_* < 43.64	0.56	0.39	0.2
		density ratio*_mean_* >= 0.9195			
		echo ratio*_mean_* < 0.056			

	branch3: veg	amplitude*_mean_* >= 43.64	0.75	0.36	0.72
		echo ratio*_mean_* >= 0.391			

	branch4: veg	amplitude*_mean_* < 43.64	0.1	0.13	0.13
		density ratio*_mean_* >= 0.9195			
		echo ratio*_mean_* >= 0.056			

	branch5: veg	amplitude*_mean_* < 43.64	31.52	12.8	25.24
		density ratio*_mean_* < 0.9195			

**Table 4.** Error assessment based on point statistics (*RP* = training area *Rathauspark, BG* = validation site 1 *Burggarten, V G* = validation site 2 *V olksgarten*).

	*RP*	*BG*	*VG*

**Number of**			
total points	549,944	559,963	537,945
points (outlier removed)	549,330	559,784	537,882
non-vegetation points in reference	374,435	393,282	463,225
vegetation points in reference	175,509	166,681	74,720

**Overall accuracy** [%]			
*CT_ew_ c_p_*=0.01	96.44	96.75	97.84
*CT_ew_ c_p_*=0.004	97.23	96.52	97.73
*CT_ampl_*	97.90	94.18	98.09

**Average accuracy** [%]			
*CT_ew_ c_p_*=0.01	97.10	96.96	95.19
*CT_ew_ c_p_*=0.004	97.57	97.18	95.19
*CT_ampl_*	97.80	91.26	95.13

**Correctness (class vegetation)** [%]			
*CT_ew_ c_p_*=0.01	91.04	92.15	92.90
*CT_ew_ c_p_*=0.004	93.52	90.48	92.06
*CT_ampl_*	97.53	95.97	95.12

**Completeness (class vegetation)** [%]			
*CT_ew_ c_p_*=0.01	98.92	97.48	91.52
*CT_ew_ c_p_*=0.004	98.51	98.81	91.66
*CT_ampl_*	97.53	84.07	91.03

## Footnotes

1 http://www.wien.gv.at/stadtentwicklung/stadtvermessung

2 http://www.diamond-air.at/airbornesensing.html

3 http://www.area-vermessung.at

4 http://www.riegl.com

5 A manual reference classification of laser scanner data in combination with orthophotos is used by Sithole and Vosselman [2] to evaluate the derivation of DTMs.

6 http://www.inpho.de
